# Improvement of Tactile Discrimination Performance and Enlargement of Cortical Somatosensory Maps after 5 Hz rTMS

**DOI:** 10.1371/journal.pbio.0030362

**Published:** 2005-10-18

**Authors:** Martin Tegenthoff, Patrick Ragert, Burkhard Pleger, Peter Schwenkreis, Ann-Freya Förster, Volker Nicolas, Hubert R Dinse

**Affiliations:** **1** Department of Neurology, Ruhr-University Bochum, BG-Kliniken Bergmannsheil, Bochum, Germany,; **2** Institute for Neuroinformatics, Department of Theoretical Biology, Ruhr-University Bochum, Bochum, Germany,; **3** International Graduate School of Neuroscience, Ruhr-University Bochum, Bochum, Germany,; **4** Department of Radiology, Ruhr-University Bochum, BG-Kliniken Bergmannsheil, Bochum, Germany; National Institute of Neurological Disorders and StrokeUnited States of America

## Abstract

Repetitive transcranial magnetic stimulation (rTMS) is increasingly used to investigate mechanisms of brain functions and plasticity, but also as a promising new therapeutic tool. The effects of rTMS depend on the intensity and frequency of stimulation and consist of changes of cortical excitability, which often persists several minutes after termination of rTMS. While these findings imply that cortical processing can be altered by applying current pulses from outside the brain, little is known about how rTMS persistently affects learning and perception. Here we demonstrate in humans, through a combination of psychophysical assessment of two-point discrimination thresholds and functional magnetic resonance imaging (fMRI), that brief periods of 5 Hz rTMS evoke lasting perceptual and cortical changes. rTMS was applied over the cortical representation of the right index finger of primary somatosensory cortex, resulting in a lowering of discrimination thresholds of the right index finger. fMRI revealed an enlargement of the right index finger representation in primary somatosensory cortex that was linearly correlated with the individual rTMS-induced perceptual improvement indicative of a close link between cortical and perceptual changes. The results demonstrate that repetitive, unattended stimulation from outside the brain, combined with a lack of behavioral information, are effective in driving persistent improvement of the perception of touch. The underlying properties and processes that allow cortical networks, after being modified through TMS pulses, to reach new organized stable states that mediate better performance remain to be clarified.

## Abstract 2

Functional magnetic resonance imaging and sensory threshold testing demonstrate that brief periods of transcranial magnetic stimulation can induce changes in somatosensory processing.

## Introduction

Repetitive transcranial magnetic stimulation (rTMS) is increasingly used both as a tool to explore the mechanisms and consequences of cortical plasticity in the human brain and as a new therapeutic strategy. During a typical rTMS experiment, an electromagnetic coil is placed above the scalp. The coil produces magnetic pulses that pass through the skull and induce electric currents within the brain that alter the activity of the underlying neurons (for review see [1–4]). In a way, rTMS can be regarded as a form of an in-vitro long-term potentiation/long-term depression (LTP/LTD) protocol, because both approaches share the aspect of passive unattended stimulation in combination with a lack of task and behavioral information. The fact that brain activation is induced from outside the skull allows for full control of the region of the brain being stimulated. Besides offering exciting new approaches to revealing basic mechanisms of brain function, recent studies suggest that rTMS might also be useful as a therapeutic tool. For example, rTMS appears to be effective in improving the mood of people with depression [5–7].

Generally, rTMS effects depend on intensity and frequency, and there is agreement that low-frequency rTMS, usually 1 Hz or lower, leads to cortical suppression, while high-frequency rTMS (5 Hz and higher) results in enhanced excitation ([8,9]; for review see [4]).

During recent years we searched for approaches that allow a better understanding of factors that are particularly effective in driving and controlling learning processes. As a first step in this direction, we have introduced tactile coactivation as a method. Coactivation follows closely the idea of Hebbian learning [10]: Synchronous neural activity, which is regarded as instrumental to driving plastic changes, is generated by the simultaneous tactile “costimulation” of skin surfaces on the tip of the index finger (also termed second digit [D2]). Accordingly, coactivation is a task-free, unattended stimulation that enforces localized activation patterns. The protocol has been shown to induce a particular form of perceptual learning that parallels cortical reorganization [11–17]. Because coactivation allows for full control of the input statistics used for driving plastic changes, it is highly effective in the systematic study of the impact of unattended but patterned stimulation on plastic capacities of cortical networks and associated perceptual skills without invoking task training or cognitive factors such as attention or reinforcement [18]. The latter is in line with a recent study, according to which perceptual learning occurs even without awareness by repetitive exposure to stimuli that are below the threshold of visibility and that are irrelevant to the central task [19].

In the present study, we went one step further: Under the assumption that coactivation induces synchronous neural activity at a cortical site selected by the location of tactile stimulation, we short-cut the entire sensory pathway to enforce locally, from outside the skull, synchronized activity over the index finger representation of primary somatosensory cortex (SI) by means of high-frequency (5 Hz) rTMS. Experiments on cortical excitability changes documented that cortical processing can be altered by means of repetitive current pulses from outside the skull. Recently we showed that 5 Hz rTMS over the median nerve representation of SI persistently increases cortical excitability [20]. However, little is known so far about high-frequency rTMS effects on perceptual skills and possible associated changes of cortical representational maps. By combining psychophysical methods with functional imaging, we demonstrate here that 5 Hz rTMS applied in humans persistently improves tactile discrimination performance as a marker of perceptual skills parallel to changes in SI maps.

## Results

### Psychophysical Effects of rTMS

We first assessed tactile acuity by measuring two-point discrimination thresholds on the tips of the right and left index finger in 33 right-handed participants (Figure 1). To obtain a stable baseline, we tested in all participants thresholds of the right index finger in five consecutive sessions that revealed little fluctuation in thresholds (multivariate ANOVA with factor session: F_[4,128]_ = 1.296, *p* = 0.275; Figure 2A). After the fifth session, rTMS was applied with a figure-eight coil positioned over the representation of the right index finger in left SI. Discrimination thresholds were retested 5 min after termination of rTMS. Psychometric functions showed a distinct shift towards smaller separation distances after rTMS, indicating that discrimination thresholds were significantly lowered (Figures 2A, 3, and 4; repeated measures of the difference between pre-rTMS and post-rTMS [hereafter referred to as pre-post] ANOVA F_[1,32]_ = 333.401, *p* < 0.0001; posthoc Scheffé-test of post-rTMS versus session (S) 1–S5 [S5 is the pre-rTMS session]; 0.003 ≥ *p* ≤ 0.0001, *n* = 33).

**Figure 1:**
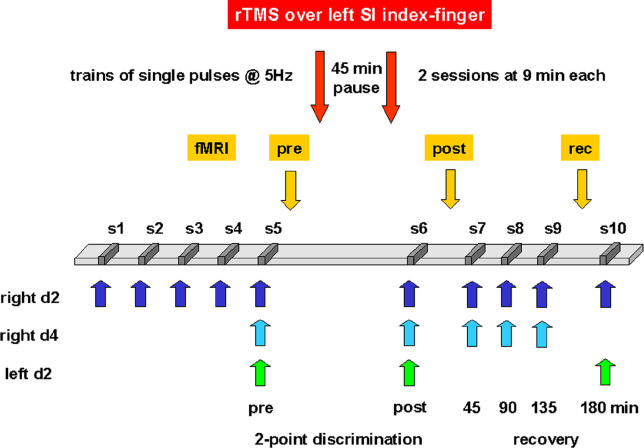
Experimental Design S1–S5 served to create a stable discrimination performance for the right D2. D4 and the left index finger (D2), serving as controls to assess spatial specificity of the effects, were tested at session S5 (pre-rTMS), because task familiarization has been shown to generalize across fingers. After S5, pre-fMRI measurements were performed, then two rTMS sessions separated by 45 min were applied over the left cortical index finger representation in SI. After the termination of rTMS, in session S6 the post-rTMS condition was measured for both index fingers and the ring finger. Then post-fMRI measurements were repeated. S7–S10 served to assess the recovery of the rTMS-induced effects on thresholds. Recovery of BOLD signals was assessed 3 h after termination of rTMS.

**Figure 2:**
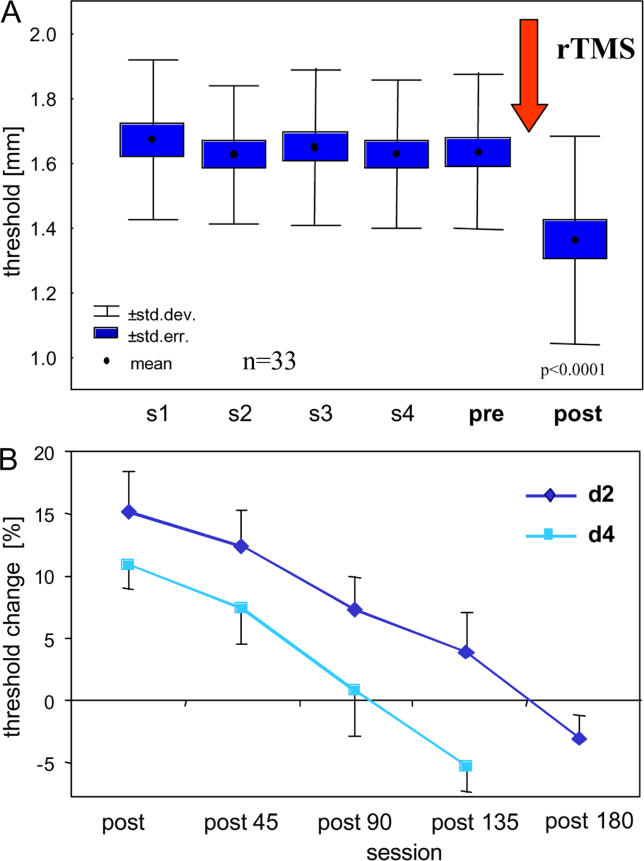
Effects of rTMS on Discrimination Thresholds (A) Effects of rTMS on discrimination thresholds on the right index finger. Data represent average from all participants (*n* = 33). Dots represent mean thresholds, boxes show the standard errors, and whiskers correspond to the standard deviation. rTMS period over of the right index finger representation in left SI is indicated by an arrow. Shown are the baseline measurements from 5 consecutive sessions before rTMS. After session S5 (pre-rTMS condition), rTMS was applied. After rTMS (post-rTMS), discrimination thresholds were significantly reduced. (B) Comparison of the rTMS-induced threshold changes of D2 and D4, and of the time course of recovery (mean ± SEM). For D4, threshold changes were smaller, and persistence of threshold changes was shorter, compared to D2, suggesting that rTMS efficacy drops over a cortical distance spanning the representations between D2 and D4.

**Figure 3:**
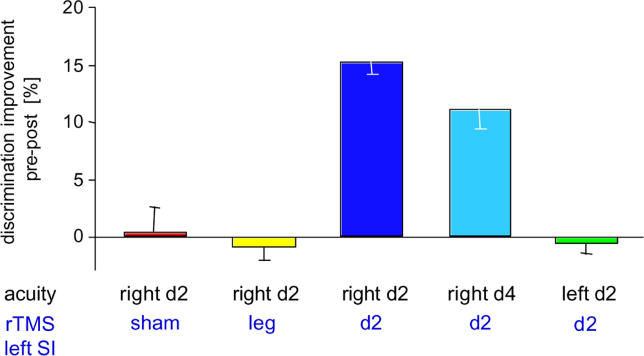
Perceptual Effects Evoked by 5 Hz TMS Mean percent changes (± SEM) in discrimination thresholds after rTMS application expressed as post-rTMS relative to pre-rTMS (pre = 100 %). At the bottom, the site of assessment of tactile acuity is given in black, and the site of TMS application is given in blue. Threshold changes are shown for the right index finger after sham rTMS (red); right index finger after rTMS over left representation of right lower leg (yellow); right index finger after rTMS over left representation of right index finger (dark blue); right ring finger after rTMS over left representation of index finger (light blue); and left index finger after rTMS over left representation of right index finger (green).

### Time Course of Recovery of Psychophysical rTMS Effects

To obtain information about the duration of the effect, we retested thresholds of the right index finger 45, 90, and 135 min after termination of rTMS (hereafter termed recovery after 45 min [rec45], recovery after 90 min [rec90], and recovery after 135 min [rec135]) in five individuals, and after 3 h in an additional six. As shown in Figure 2B, there was a gradual restoration of previous levels of performance found under pre-rTMS conditions (pre-post difference ± standard error of the mean [SEM], 0.24 mm ± 0.01; repeated measures ANOVA, F_[1,4]_ = 31.00, *p* = 0.005). These results demonstrate that tactile discrimination performance can be modified by rTMS, the improvement persisting approximately 2 h (*t-*test for D2: pre-rTMS versus rec135: *p* = 0.29, *n* = 5; *t-*test for D2: pre-rTMS versus rec180: *p* = 0.15, *n* = 6).

### Spatial Selectivity of rTMS Effects

A number of tests were performed to measure the effect of rTMS on digits other than the index finger to determine how selective tactile discrimination can be modified. Previous studies had shown that the left index finger showed a stable performance comparable to that observed for the right index finger during the initial sessions, S1–S5 [11–15,21]. As a first control of the spatial specificity, we therefore measured thresholds of the index finger of the left hand at S5 (pre-rTMS) and S6 (post-rTMS) with rTMS still applied over the right index finger representation. After rTMS (S6), thresholds of the left index finger remained unchanged (*t-*test, *p* = 0.644, *n* = 33 [Figure 3]). As a next step, we measured the discrimination thresholds of the ring finger of the right hand with rTMS delivered over the cortical representation of the right index finger. In the cortical map of the homunculus, the ring finger is located medially from the representation of the index finger. According to magnetoencephalographic mapping studies, the representations of the index finger and ring finger (also termed D4) are separated by several millimeters [22–24]. Comparing the thresholds for the ring finger pre- and post-rTMS revealed that thresholds were also lowered (pre-post difference, 0.17 mm; *t-*test, *p* = 0.004, *n* = 5) , but to a lesser extent (Figures 2B and 3). When we measured the duration of the effect of rTMS on discrimination thresholds of the ring finger, we found that the rTMS-evoked reduction of thresholds vanished within 90 min (*t-*test for D4; pre-rTMS versus rec90: p = 0.78, Figure 2B).

The spatial specificity of the rTMS effects was further tested in additional five participants, where we applied rTMS over the lower leg representation [25], but measured discrimination thresholds for the left and right index finger. Mean ± SEM of thresholds were 1.40 ± 0.08 mm for the right and 1.51 ± 0.15 mm for the left index finger before rTMS. No changes in thresholds of the index finger were found (*t-*test right D2, pre-post, *p* = 0.828; *t-*test left D2, pre-post: *p* = 0.253 [Figure 3]). Finally, we applied 5 Hz sham-rTMS over the left SI in another five participants, revealing no changes in tactile performance for the right and left index finger (*t-*test right D2, pre-post, *p* = 0.711; *t-*test left D2, pre-post, *p* = 0.729 [Figure 3]).

### Cortical Effects of rTMS

To investigate parallel cortical changes evoked by rTMS, we measured in a subpopulation of participants (*n* = 12) blood oxygen level-dependent (BOLD) signals before and after rTMS following electrical stimulation of the right and left index finger (Figure 4). The psychophysical performance of these individuals matched that described for the whole population above for both the pre- and post-rTMS condition. For the pre-rTMS session, the results of the group analysis revealed focused SI activation in the postcentral gyrus as seen in the estimated statistic parametric map (SPM) (Figure 4A). In addition, in line with previous experiments [14], we found a broad secondary somatosensory cortex (SII) activation contralateral and ipsilateral to the stimulated index finger in the parietal operculum above the Sylvian fissure (Figure 4A). For each participant, the coordinates of maximal BOLD activity could be assigned to SI or SII according to the Talairach atlas [26]. After rTMS, we found an enlargement of activation pattern and an enhancement of the amplitude of the BOLD signal only in the left, rTMS-treated SI (Figures 4A, 4C–4E, and 5). This observation was confirmed by a random-effect analysis, according to which significantly activated areas were only localized in SI ipsilateral to the rTMS-stimulated index finger representation, but not in contralateral SI or SII (Figure 4C–4E).

**Figure 4:**
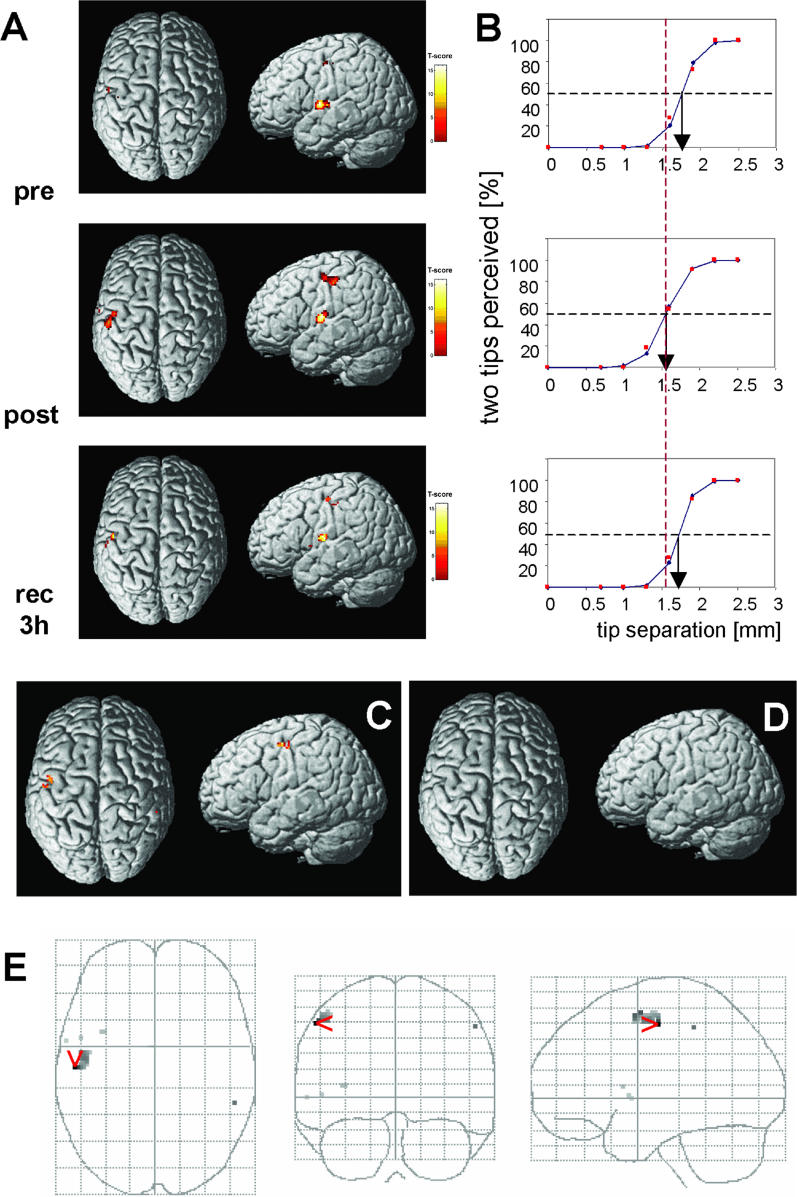
Effects of rTMS on BOLD Signals (A) rTMS effect on BOLD signals of a single participant detected pre-rTMS, post-rTMS, and 3 h after rTMS in the left SI ipsilateral to the rTMS site in the postcentral gyrus, and in the contralateral SII in the parietal operculum above the Sylvian fissure. Activations are projected on a rendered T1-weighted MRI dataset. Comparing pre- with post-rTMS fMRI sessions revealed enlarged activation and increased BOLD signal intensity in left SI ipsilateral to the rTMS site. These changes of BOLD signal characteristics recovered 3 h after termination of rTMS. (B) Psychometric functions illustrating the rTMS-induced improvement of discrimination threshold for the individual shown in (A). Correct responses in percent (red squares) are plotted as a function of separation distance together with the results of a logistic regression line (blue with blue diamonds). 50% levels of correct responses are shown as well as thresholds. Top graph, pre-rTMS; middle graph, post-rTMS condition, immediately after rTMS; bottom graph, recovery after 3 h. After rTMS there is a distinct shift in the psychometric functions towards lower separation distances by 0.20 mm, which recovers to pre-rTMS conditions 3 h later (pre-rTMS, 1.75 mm; recovered, 1.72 mm). (C) Random-effect analysis (paired *t-*test pre-post, right D2 stimulation) revealed significant changes of activated patterns localized in SI ipsilateral to the rTMS stimulated D2 representation (*n* = 12, pre- versus post-rTMS; threshold, *p* = 0.001, uncorrected for multiple comparisons; S1-parameters, 32 voxels; T-score = 4.15; x,y,z (mm), −54, −14, 50; Talairach position, postcentral gyrus, Brodmann area 3). (D) No changes of BOLD activity were found in the right hemisphere contralateral to the rTMS site (paired *t-*test pre- versus post-rTMS, left index finger stimulation; threshold, *p* = 0.001, uncorrected for multiple comparisons) and in SII. (E) Changes in activation pattern as obtained from random effects analysis (paired *t-*test pre- versus post-rTMS, right index finger stimulation; compare with [C]), superimposed on a glass brain for visualization. Views are from top (left grid), back (middle grid), and right (right grid).

According to the psychophysical data, the rTMS-induced threshold changes recovered after about 2 h. Therefore, we also assessed cortical recovery by measuring BOLD signals 3 h after termination of rTMS (*n* = 6). In all participants, the rTMS-induced changes of the right index finger came back to pre-rTMS conditions both perceptually and cortically (Figures 2B and 4A). We also recorded BOLD signals under the control conditions described above to demonstrate the spatial selectivity of the rTMS-induced changes. The cortical representations of the left index finger were unaffected after rTMS (Figure 5; *n* = 12). Similarly, imaging the right index finger after rTMS applied over the lower leg representation revealed no alterations (Figure 5; *n* = 5), as was the case after sham-rTMS (Figure 5; *n* = 5).

**Figure 5:**
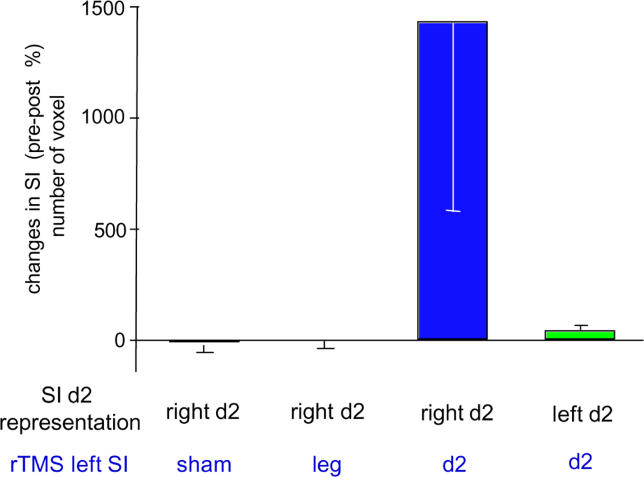
Cortical Effects Evoked by 5 Hz TMS Mean percent changes (± SEM) of the number of activated voxels after rTMS application expressed as post-rTMS relative to pre-rTMS (pre = 100 %). At the bottom, the site of cortical measurement (cortical representation of the index finger D2 in SI) is given in black, and the site of TMS application is given in blue. Shown are cortical changes in activation of the representation of the right index finger in left SI after sham rTMS (red); right index finger in left SI after rTMS over the lower leg representation in left SI (yellow); right index finger in left SI after rTMS over the index finger representation in left SI (blue); and left index finger in right SI after rTMS over the index finger representation in left SI (green).

### Relation between rTMS-Induced Discrimination Improvement and Reorganization

The rTMS-induced effects on discrimination thresholds all varied individually, as did the enlargement of the right index finger representations. According to animal studies, map size changes are a reliable predictor of changes in individual performance [27,28]. We therefore performed a linear correlation analysis (Pearson) between cortical enlargement (number of activated voxels) and psychophysical threshold reduction [13–16], which revealed a significant relationship between the rTMS-induced map enlargement and parallel changes in discrimination thresholds (Figure 6). Individuals who showed a large cortical reorganization benefited most from rTMS, while little gain in discrimination abilities was associated with small changes in BOLD signals (Pearson linear correlation, *r* = 0.603, *p* = 0.037). This analysis indicates a close link between individual gain in performance and the degree of reorganizational changes, which was restricted to the contralateral SI digit representation.

**Figure 6:**
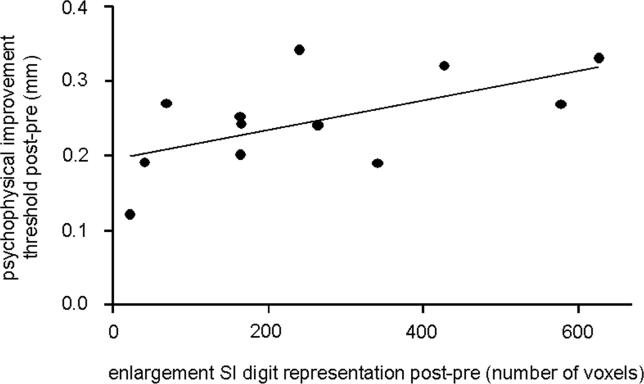
Relation between Perceptual and Cortical rTMS-Evoked Changes Linear correlation analysis (Pearson, *r* = 0.603, *p* = 0.037) between individual rTMS induced changes in BOLD signals in SI (difference in activated voxels post-pre) and associated changes of two-point discrimination thresholds post-pre.

## Discussion

Our results demonstrate that an improvement of tactile discrimination of the index finger can be induced directly by applying 5 Hz rTMS to the cortical index finger representation of SI from outside the skull. The increase in perceptual performance persisted up to 2 h after termination of rTMS, and was accompanied by an enlargement of the index finger representation in SI. The cortical changes had the same time course of recovery as the perceptual improvement. Most notably, the individual degree of cortical enlargement was correlated with the individual gain in tactile acuity induced by rTMS. Thus, to our knowledge this is the first report that a locally enforced cortical activation pattern, evoked by applying 5 Hz rTMS over the index finger representation in SI (a procedure that shortcuts the entire sensory pathway), has the potential to drive perceptual improvements that outlast the stimulation period for almost 2 h. The underlying processes are spatially specific and can be assessed psychophysically as well as by means of noninvasive imaging techniques.

### Spatial Selectivity of the Observed Effects

In order to drive specific changes in perception, the underlying cortical activation induced by rTMS should be specific. Evidence for spatially far-reaching alterations has been demonstrated with low-frequency rTMS. Enhancement of excitability in the primary motor cortex (MI) was described after 1 Hz rTMS in contralateral MI that persisted for 30 min through a modulation of interhemispheric interactions [29]. The same conclusion was reached in a study demonstrating improvement of ipsilateral sequential simple finger movements that persisted for 10 min after 1 Hz rTMS over MI, presumably by releasing the contralateral MI from transcallosal inhibition [30].

We therefore performed a number of control experiments designed to provide information about the spatial specificity, i.e., skin areas that underwent changes in discrimination. Specifically, we compared discrimination performance of the right and left index finger after rTMS over the index finger representation in left SI, and performance of the right index and ring finger after rTMS over the index finger representation in left SI. In addition, we compared performance of the right index finger after rTMS was applied over the representation of the index finger or lower leg (Figures 3 and 5).

Generally, we found no effects when the test site (left index finger) and the rTMS-treated site (right index finger representation) were within different hemispheres. More importantly, we also found no effects when the test site (index finger) and the rTMS-treated site (lower leg representation) were within the same cortical body map of the homunculus in left SI, but were separated by several centimeters [22,23]. This lack of effects indicates that applying rTMS at a more remote location resulted in no recordable changes of the index finger, neither for the psychophysical assessment of discrimination nor for the imaging of BOLD signals. This finding is compatible with recent positron emission tomography imaging data that demonstrated that the region of tissue that is activated during 5Hz rTMS is confined and focused [31].

After rTMS application over the index finger representation, we observed a discrimination improvement for the index and the ring finger, which are separated cortically by only a few millimeters [22–24]. However, the rTMS effects on the threshold of the ring finger were smaller in magnitude and were observable for a shorter period of time after rTMS as compared to the index finger, which supports the argument that there is a drop-off of rTMS efficacy over a cortical distance spanning the representations between the index and ring fingers. An even more refined spatial effectiveness of rTMS might be limited not by the spread of rTMS currents but by a general limitation of interdigit separation. In fact, presenting vibrotactile spatial patterns in a pattern identification task revealed substantial interference across fingers of the same hand based on response competition [32]. The typical pattern of mislocalizations onto neighboring fingers observed in a finger localization task can best be explained by digit-overlapping receptive fields [33]. Evidence for considerable cross-talk between neighboring digits comes from the above coactivation experiments described in the introduction, where only the tip of the index finger is coactivated: While the index finger of the left hand remains unaffected in all cases, the tactile two-point discrimination thresholds were also lowered on neighboring digits D1 and D3 (unpublished data).

### Forms of Unattended Stimulation

The major finding of our study—a demonstration of perceptual improvement as a marker of synaptic alterations solely through unattended stimulation from outside the skull—is not restricted to SI. Using an orientation discrimination task [34], we recently demonstrated that 5 Hz rTMS applied over central primary visual cortex improves orientation discrimination in humans for 24 h [35].

Besides low- and high-frequency rTMS, the excitability of the cerebral cortex can be modulated by transcranial direct current stimulation (tDCS), which offers the advantage of portable equipment. In addition to excitability changes, which can be controlled by the polarity, duration, and current strength of stimulation [36], tDCS also affects perception, such as a suppression of tactile discrimination through cathodal stimulation [37]. Recent findings in a patient with chronic subcortical ischemic stroke document the beneficial effect of tDCS on motor function and raise the hypothesis of its potential application in neurorehabilitation [38].

A variant of rTMS resembling our rTMS protocol is the so-called intracortical microstimulation (ICMS), which is used in animal models to drive reorganization of cortical and subcortical maps and receptive fields [18,39–43]. ICMS consists of high-frequency electrical pulse trains that are delivered through a microelectrode at very low currents, usually less than 10 μA [39,40,44]. ICMS has been interpreted as a form of LTP/LTD-like stimulation applied under in vivo conditions in the intact brain [40,45]. Because both ICMS and rTMS enforce neural activity locally, both protocols work largely independently of the peripheral and subcortical pathways and the constraints provided by particularities of a sensory pathway and its preprocessing.

### Relation of rTMS Effectiveness to Coactivation

As outlined in the introduction, application of the so-called coactivation protocol in humans revealed its substantial potential to alter tactile performance in parallel to cortical enlargement [12–16,21,46]. During the coactivation, skin locations on the tip of the index finger are simultaneously coactivated to induce temporally synchronized activation in the cortical finger representation, and rTMS shortcuts the sensory pathway by directly enforcing temporally structured activity at cortical sites.

The common idea behind both protocols is to induce changes of cortical representations solely through unattended, passive stimulation [46]. At a psychophysical level, both protocols evoked the same magnitude of changes in tactile acuity thresholds; however, rTMS-induced changes recovered faster (3 h after rTMS versus 24 h after coactivation). Another difference was found for the associated changes in cortical representations: While both protocols increased the size of the cortical finger representation in SI, where the amount of reorganization was linearly correlated with the gain in discrimination improvement, rTMS did not affect the finger representation in SII as compared to coactivation. One explanation for this discrepancy could be that SII reorganization requires either direct activation or activation mediated through ascending subcortical pathways [47].

The rTMS results presented here, in combination with those of the coactivation and ICMS experiments, demonstrate that unattended stimulation protocols alone are sufficient to drive plastic changes when they fulfill the requirement of high-frequency repetition, as in the case of rTMS or ICMS, or the requirement of simultaneity, i.e., pairing of stimuli. Cortical excitability has been shown to be modulated by subtle adjustment of the timing of a paired associative stimulation protocol of repetitively pairing median nerve simulation with TMS over human motor cortex [48]. The importance of simultaneity in the coactivation task was demonstrated by using a single, small stimulation site instead of one large area. When this protocol was applied for 3 h, no effects on discrimination nor on cortical maps in SI and SII were found, indicating that “coactivation” is indeed crucial [14].

### Relation between Cortical Excitability Changes and Perception

Effects of rTMS are often described in terms of perceptual extinction, a so-called virtual lesion [2,3], and in terms of changes of cortical excitability. Low-frequency stimulation, usually in the range of 1 Hz, has been shown to induce inhibition [9,49,50], while so-called “high-frequency” stimulation (around 5 Hz or higher) leads to increased excitability [8,51–54]. Interestingly, changes in cortical excitability are not limited to the period of stimulation, but can persist for variable amounts of minutes.

Perceptually, the detection of tactile stimuli is suppressed during rTMS [55], as is localization performance [56]. Using low-frequency rTMS, impairment of tactile frequency discrimination performance in humans has been reported that outlasted the stimulation period by maximally 10 min, while the duration of impairment was correlated with the duration of rTMS application [57]. Similar short-lasting effects on tactile performance were described after 0.9 Hz rTMS applied over sensorimotor cortex [58]. These authors reported a transient increase of touch thresholds for several minutes, but no changes in two-point discrimination or median nerve somatosensory evoked potentials. Given the low frequency rTMS-induced effects on cortical excitability through increased inhibition, the reported impairment of tactile performance might be related to the LTD-like processes evoked. In contrast, low-frequency rTMS effects appear to be more complex. In the language domain, it was recently shown that 1 Hz rTMS can temporarily impair or improve language processing, depending on the cortical area stimulated [59].

Signatures of LTP-like processes include an enhancement of cortical excitability. Using a paired pulse protocol of median nerve stimulation, we have recently demonstrated that, after applying the same 5 Hz rTMS protocol as described here, enhanced cortical excitability persisted for more than 1 h, as indicated by a reduced paired pulse inhibition recorded over SI median nerve representation [20]. Accordingly, the described improvement of two-point discrimination performance after 5 Hz rTMS was probably based on processes that involve increased cortical excitability.

### Excitability Versus Learning

Are our findings a mere consequence of excitability changes, or do they represent a form of learning? At a cellular level, the use of electrical stimulation of defined temporal statistics is surprisingly effective to induce persistent changes in synaptic transmission. Depending on the frequency of stimulation, specific forms of long-term changes of synaptic efficacy can be established such as LTP or LTD [60–62]. Although stimulation protocols of that kind are unrelated to any task, and do not require forms of attention, reinforcement, training or behavioral information, the results derived from such studies were instrumental in providing insight not only into mechanisms of plasticity, but also into processes related to learning and memory [63,64]. Recently it was demonstrated that a classic theta burst stimulation protocol used to induce LTP/LTD in brain slices can be adapted to a TMS protocol to rapidly produce long-lasting (up to 1 h), reversible effects on motor cortex physiology and behavior [65]. Accordingly, we suggest that the outcome of the 5 Hz TMS application represents a form of learning rather than the consequence of cortical excitability changes.

We conclude that 5 Hz rTMS results in a restructuring of the stimulated cortical networks, which leads to changes in synaptic efficacy and cortical excitability, which in turn modifies the way sensory information is processed. Most notably, these changes do not leave the network in a disorganized state, but on the contrary lead to the emergence of a different, yet organized and meaningful behavior as indicated by the improvement of discrimination performance. What (among other issues) remains to be clarified are the underlying properties and processes that enable cortical networks to stabilize after being modified through TMS pulses without behavioral or contextual information into new organized states mediating better performance.

Our results add to the view that primary sensory areas play a crucial role in perceptual learning [66–69]. Recently, single-pulse TMS has been shown to disrupt a working memory task of frequency discrimination in humans [70]. These results were taken as an indication that the primary sensory cortex seems to act not only as an on-line processor but also as a transient storage site for information that contributes to working memory [70]. Our present findings go one step further: By applying a stimulation protocol of rTMS (which resembles those used in brain slices to induce synaptic plasticity) presented from outside directly to selected brain regions, we can induce plasticity processes that resemble learning in some key aspects. As a result, cortical representations in human SI enlarge, thereby improving individual perceptual abilities.

## Materials and Methods

### Experimental schedule

A total of 49 right-handed individuals (19 males; mean age ± standard deviation = 31.6 ± 3.7 y) participated in this study, which was performed in accordance with the Declaration of Helsinki. For an overview of participants' assignments to different tests, see Table 1. All participants gave their written informed consent, and the protocol was approved by the local ethical committee of the Ruhr-University Bochum. In most experiments (see below), the cortical representation of the index finger of the right hand in the left SI was used for rTMS. To obtain a stable baseline of discrimination, we tested the participants on five consecutive sessions on the right index finger (see Figure 1). Sessions were statistically analyzed for stability (ANOVA). In the fifth session, the thresholds of the left index finger were additionally measured. Previous studies had shown that this initial task familiarization completely generalizes to the other fingers [11–15]. After assessment of thresholds of both fingers (pre-rTMS condition), participants were subjected to functional magnetic resonance imaging (fMRI) measurements to obtain the pre-rTMS activation pattern. Then rTMS was applied. Discrimination performance of the index fingers was retested starting about 5 min after the termination of rTMS (post-rTMS condition). Then fMRI measurements were repeated to measure post-rTMS activation pattern. Recovery of threshold was assessed 45, 90, and 135 min after termination of rTMS. Recovery of BOLD effects was measured 3 h after termination of rTMS. Additional controls consisted of measuring discrimination thresholds for the ring finger, measuring BOLD activity and thresholds of each index finger after rTMS over the lower leg representation, or after sham stimulation.

**Table 1:**
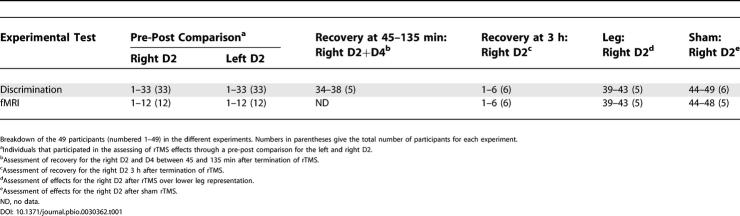
Summary of Experiment Participants

### Measurement of two-point discrimination thresholds

Tactile two-point discrimination thresholds of the tips of the index fingers were assessed using the method of constant stimuli as described previously [11–13,15]. Seven pairs of needles (diameter 200 microns) separated by 0.7, 1.0, 1.3, 1.6, 1.9, 2.2, and 2.5 mm were used. In addition, zero distance was tested with a single needle. The participants had been instructed that there were single needle presentations for control, but not how often. No feedback was given. The needles were mounted on a rotatable disc that allowed rapid switching between distances. To accomplish a uniform and standardized type of stimulation, the disc was installed in front of a plate that could be moved up and down. The arm and fingers of the participants were fixated on the plate, and participants were then asked to move the arm down. The down-movement was arrested by a stopper at a fixed position above the needles. The test finger was held in a hollow containing a small hole through which the finger touched the needles at approximately the same indentations in each trial. Each distance of the needles was tested ten times in randomized order, resulting in 80 single trials per session. The participant had to decide immediately whether the sensation was of one or two tips by answering “one” or “two.” The summed responses were plotted against distance as a psychometric function for absolute threshold, fitted by a binary logistic regression (SPSS, Chicago, Illinois, United States). Threshold was taken from the fit at the distance where 50% correct responses was reached.

### Repetitive rTMS

A MAGSTIM Rapid Stimulator (Magstim, Whitland, Dyfed, UK) connected to an eight-shaped coil was used for application of rTMS [20,71]. During the rTMS sessions, participants were seated in a comfortable chair and wore a tight-fitted cap with a 1-cm grid referenced to the vertex (Cz). Motor thresholds (MTs) were measured at the relaxed first dorsal interosseous (FDI) muscle of the right hand using single-pulse TMS [20,46,71]. The FDI representation was identified at the position at which TMS induced highest motor-evoked potentials (MEPs). MT was defined as the lowest intensity capable of evoking five out of ten MEPs with an amplitude of at least 50 μV. To position the coil over the right index finger representation in the left SI, the coil was moved in accordance with Maldjian et al. [24], 1–2 cm posterior in parasagittal direction to a position, where participants reported detectable sensations in their right index finger induced by single pulse TMS (intensity 90% of the MT) such as tickling or prickling [71]. Sensations such as tingles evoked by direct activation of SI might be analogous to phosphenes, which can be induced by direct activation of visual cortex [72]. However, although the focus of stimulation was clearly remote from MI, direct or indirect influences from MI activation can not be completely ruled out. rTMS intensity was set at 90% of the MT. During subsequent rTMS stimulation, surface electromyograms were recorded from the right FDI muscle. The spatial specificity of the rTMS effects was tested by applying rTMS over the right lower leg representation, using a similar localization as described above. First, the tibialis nerve representation was identified at the position at which single-pulse TMS induced highest evoked MEPs 1–2 cm lateral to Cz. To position the coil over the right lower leg representation in the left SI, the coil was moved 1–2 cm posterior in a parasagittal direction to a position where participants reported detectable sensations in their right lower legs induced by single-pulse TMS. rTMS intensity was set at 90% of the MT. During subsequent rTMS stimulation, surface electromyograms were recorded from the right tibial anterior muscle, while discrimination thresholds for both index fingers were measured.

TMS pulses in 25 trains were applied through the tangentially oriented coil with the grip pointing backward positioned over SI [20,71]. A train consisted of 50 single pulses of 5 Hz lasting 10 s with an intertrain interval of 5 s. Five consecutive trains were grouped into one block. Between two subsequent blocks, a rest period of 1 min was interposed. Next, 45 min after the termination of this session, rTMS was repeated in a second session, with stimulation intensity, magnetic coil position, and parameter settings kept constant, resulting in a total of 2,500 TMS pulses. In the sham-rTMS condition, the coil was tilted 45° off the surface of the head, while only the edge of the coil touched the scalp. Generally, rTMS application was well tolerated in all participants, and no side effects could be observed.

### fMRI scanning

fMRI measurement was performed with a whole-body 1.5 T scanner (Magnetom Symphony, Siemens, Germany) equipped with a high-power gradient-system (30 mT/m/s; SR 125 T/m/s), using a standard imaging head coil. Procedures were the same as those described recently by Pleger and coworkers [14]. Generally, we used a block design without requiring the participants to perform any tasks during scanning. Behavioral testing was done separately before and after scanning. BOLD images were obtained with a single-shot SpinEcho-EPI sequence (TR 1,600 ms; TE 60 ms; matrix 64 × 64; FOV 224 mm; slice thickness 5 mm; gap between slices 1 mm; voxel 3.5 mm × 3.5 mm × 5 mm). We acquired 16 transaxial slices parallel to the AC-PC line, which covered the whole brain excluding cerebellum. For finger stimulation we used a TENS stimulator (Medicommerz, Kirchzarten, Germany) with conventional ring-electrodes mounted on the tip of the index finger (pulse duration 0.1 ms; repetition rate 3 Hz; stimulation intensity 2.5 times above threshold). Electrodes were removed between sessions, but position on the fingers were marked. Each activation study comprised nine blocks of rest and eight blocks of stimulation, each of which contained 40 scans. BOLD activation after electrical stimulation of the index finger of each hand was measured in separate sessions that were counterbalanced. Anatomical images were acquired using an isotropic T1-3dGE (MPRAGE) sequence (TR 1,790 ms; TE 388 ms; matrix 256 × 256; field of view 256 mm; slice thickness 1 mm; no gap; voxel size 1 mm × 1 mm × 1 mm) with 160 sagittally oriented slices covering the whole brain.

### Data analysis

For preprocessing and statistical analysis of the fMRI data, we used the Statistical Parametric Mapping software package, version 99 (MATLAB, Mathworks, Natick, Massachusetts, United States). The first ten images of each fMRI session (690 images) were discarded from further analysis. First, all scans were realigned, and a mean image was formed in the process. Scans were resliced using Sinc interpolation. The individual three-dimensional datasets were normalized using the standard template of the Montreal Neurological Institute (MNI) (voxel size, 2 mm^3^) to establish Talairach coordinates from the MNI data (http://www.mrc-cbu.cam.ac.uk/Common/People/people-pages/Matthew.Brett.shtml). Scans were smoothed with a 6-mm (full-width half-maximum) isotropic, three-dimensional Gaussian filter. Statistical maps were calculated using a high-pass cut-off at 256 s, a hemodynamic response function (lowpass filter) and a threshold of *p* = 0.05 (corrected for multiple comparisons). We calculated Statistic parametric maps (first-level analysis) for each individual and for each session using *t-*contrasts (*t-*test) of SPM contrast manager. For the topographic assignment of BOLD signals of the different measurements, we co-registered the mean image formed in a realignment procedure with the anatomic image from the T1-GE sequence scan. Generally, no region of interest analysis was used. Statistical parametric maps of group analysis were generated using the contrast files of the first-level analysis to identify localization, cluster-level, MNI coordinates, and size of activated patterns.

To evaluate differences of pre- and post-rTMS sessions, we performed a random-effect analysis using Student's paired *t-*test of right and left sides separately. Significance was determined using *p* = 0.001 for peak height and uncorrected for multiple comparisons. The effect of rTMS on BOLD signals was expressed by post-pre difference maps for each individual. To analyze the relationship between rTMS-induced changes in BOLD signals and the individual perceptual changes in discrimination thresholds, we performed a simple correlation analysis. The differences in thresholds before and after rTMS (post-pre) were inserted as covariates. Linear correlation analysis (Pearson) between individual pre- and post-rTMS maps were estimated using equal thresholds for all sessions (*p* = 0.05; corrected for multiple comparisons).

## Abbreviations

BOLD: blood oxygenation level-dependent
D: digit
FDI: first dorsal interosseous
fMRI: functional magnetic resonance imaging
ICMS: intracortical microstimulation
LTD: long-term depression
LTP: long-term potentiation
MEP: motor-evoked potential
MI: primary motor cortex
MNI: Montreal Neurological Institute
MT: motor threshold
rTMS: repetitive transcranial magnetic stimulation
S: session
SEM: standard error of the mean
SI: primary somatosensory cortex
SII: secondary somatosensory cortex
SPM: statistic parametric map
tDCS: transcranial direct current stimulation

## References

[pbio-0030362-b01] Grafman J, Wassermann E (1999). Transcranial magnetic stimulation can measure and modulate learning and memory. Neuropsychologia.

[pbio-0030362-b02] Pascual-Leone A, Walsh V, Rothwell J (2000). Transcranial magnetic stimulation in cognitive neuroscience—Virtual lesion, chronometry, and functional connectivity. Curr Opin Neurobiol.

[pbio-0030362-b03] Pascual-Leone A, Bartres-Faz D, Keenan JP (1999). Transcranial magnetic stimulation: Studying the brain-behaviour relationship by induction of “virtual lesions.”. Philos Trans R Soc Lond B Biol Sci.

[pbio-0030362-b04] Siebner HR, Rothwell J (2003). Transcranial magnetic stimulation: New insights into representational cortical plasticity. Exp Brain Res.

[pbio-0030362-b05] Klein E, Kreinin I, Chistyakov A, Koren D, Mecz L (1999). Therapeutic efficacy of right prefrontal slow repetitive transcranial magnetic stimulation in major depression: A double-blind controlled study. Arch Gen Psychiatry.

[pbio-0030362-b06] Kauffmann CD, Cheema MA, Miller BE (2004). Slow right prefrontal transcranial magnetic stimulation as a treatment for medication-resistant depression: A double-blind, placebo-controlled study. Depress Anxiety.

[pbio-0030362-b07] Pascual-Leone A, Rubio B, Pallordo F, Catala MD (1996). Beneficial effect of rapid-rate transcranial magnetic stimulation of the left dorsolateral prefrontal cortex in drug resistant depression. Lancet.

[pbio-0030362-b08] Pascual-Leone A, Valls-Sole J, Wassermann EM, Hallett M (1994). Responses to rapid-rate transcranial magnetic stimulation of the human motor cortex. Brain.

[pbio-0030362-b09] Chen R, Classen J, Gerloff C, Celnik P, Wassermann EM (1997). Depression of motor cortex excitability by low-frequency transcranial magnetic stimulation. Neurology.

[pbio-0030362-b10] Hebb D (1949). The organization of behavior.

[pbio-0030362-b11] Godde B, Spengler F, Dinse HR (1996). Associative pairing of tactile stimulation induces somatosensory cortical reorganization in rats and humans. Neuroreport.

[pbio-0030362-b12] Godde B, Stauffenberg B, Spengler F, Dinse HR (2000). Tactile coactivation-induced changes in spatial discrimination performance. J Neurosci.

[pbio-0030362-b13] Pleger B, Dinse HR, Ragert P, Schwenkreis P, Malin JP (2001). Shifts in cortical representations predict human discrimination improvement. Proc Natl Acad Sci U S A.

[pbio-0030362-b14] Pleger B, Foerster AF, Ragert P, Dinse HR, Schwenkreis P (2003). Functional imaging of perceptual learning in human primary and secondary somatosensory cortex. Neuron.

[pbio-0030362-b15] Dinse HR, Ragert P, Pleger B, Schwenkreis P, Tegenthoff M (2003). Pharmacological modulation of perceptual learning and associated cortical reorganization. Science.

[pbio-0030362-b16] Hodzic A, Veit R, Karim AA, Erb M, Godde B (2004). Improvement and decline in tactile discrimination behavior after cortical plasticity induced by passive tactile coactivation. J Neurosci.

[pbio-0030362-b17] Ragert P, Schmidt A, Altenmuller E, Dinse HR (2004). Superior tactile performance and learning in professional pianists: Evidence for meta-plasticity in musicians. Eur J Neurosci.

[pbio-0030362-b18] Dinse HR, Merzenich MM (2002). Adaptation of inputs in somatosensory system. Fahle M, Poggio T, editors. Perceptual learning.

[pbio-0030362-b19] Watanabe T, Nanez JE, Sasaki Y (2001). Perceptual learning without perception. Nature.

[pbio-0030362-b20] Ragert P, Becker M, Tegenthoff M, Pleger B, Dinse HR (2004). Sustained increase of somatosensory cortex excitability by 5 Hz repetitive transcranial magnetic stimulation studied by paired median nerve stimulation in humans. Neurosci Lett.

[pbio-0030362-b21] Dinse HR, Ragert P, Pleger B, Schwenkreis P, Tegenthoff M (2003). GABAergic mechanisms gate tactile discrimination learning. Neuroreport.

[pbio-0030362-b22] Yang TT, Gallen CC, Schwartz BJ, Bloom FE (1993). Noninvasive somatosensory homunculus mapping in humans by using a large-array biomagnetometer. Proc Natl Acad Sci U S A.

[pbio-0030362-b23] Nakamura A, Yamada T, Goto A, Kato T, Ito K (1998). Somatosensory homunculus as drawn by MEG. Neuroimage.

[pbio-0030362-b24] Maldjian JA, Gottschalk A, Patel RS, Detre JA, Alsop DC (1999). The sensory somatotopic map of the human hand demonstrated at 4 Tesla. Neuroimage.

[pbio-0030362-b25] Classen J, Knorr U, Werhahn KJ, Schlaug G, Kunesch E (1998). Multimodal output mapping of human central motor representation on different spatial scales. J Physiol.

[pbio-0030362-b26] Talairach J, Tournoux P (1988). Co-planar stereotaxic atlas of the human brain.

[pbio-0030362-b27] Recanzone GH, Merzenich MM, Jenkins WM, Grajski KA, Dinse HR (1992). Topographic reorganization of the hand representation in cortical area 3b owl monkeys trained in a frequency-discrimination task. J Neurophysiol.

[pbio-0030362-b28] Recanzone GH, Schreiner CE, Merzenich MM (1993). Plasticity in the frequency representation of primary auditory cortex following discrimination training in adult owl monkeys. J Neurosci.

[pbio-0030362-b29] Schambra HM, Sawaki L, Cohen LG (2003). Modulation of excitability of human motor cortex (M1) by 1 Hz transcranial magnetic stimulation of the contralateral M1. Clin Neurophysiol.

[pbio-0030362-b30] Kobayashi M, Hutchinson S, Theoret H, Schlaug G, Pascual-Leone A (2004). Repetitive TMS of the motor cortex improves ipsilateral sequential simple finger movements. Neurology.

[pbio-0030362-b31] Siebner HR, Peller M, Willoch F, Minoshima S, Boecker H (2000). Lasting cortical activation after repetitive TMS of the motor cortex: A glucose metabolic study. Neurology.

[pbio-0030362-b32] Craig JC, Qian X (1997). Tactile pattern perception by two fingers: Temporal interference and response competition. Percept Psychophys.

[pbio-0030362-b33] Schweizer R, Braun C, Fromm C, Wilms A, Birbaumer N (2001). The distribution of mislocalizations across fingers demonstrates training-induced neuroplastic changes in somatosensory cortex. Exp Brain Res.

[pbio-0030362-b34] Schoups AA, Vogels R, Orban GA (1995). Human perceptual learning in identifying the oblique orientation: Retinotopy, orientation specificity and monocularity. J Physiol.

[pbio-0030362-b35] Klaes C, Ragert P, Jancke DE, Tegenthoff M, Dinse HR (2003). rTMS induced improvement of human orientation discrimination. Soc Neurosci Abstr.

[pbio-0030362-b36] Nitsche MA, Fricke K, Henschke U, Schlitterlau A, Liebetanz D (2003). Pharmacological modulation of cortical excitability shifts induced by transcranial direct current stimulation in humans. J Physiol.

[pbio-0030362-b37] Rogalewski A, Breitenstein C, Nitsche MA, Paulus W, Knecht S (2004). Transcranial direct current stimulation disrupts tactile perception. Eur J Neurosci.

[pbio-0030362-b38] Hummel F, Cohen LG (2005). Improvement of motor function with noninvasive cortical stimulation in a patient with chronic stroke. Neurorehabil Neural Repair.

[pbio-0030362-b39] Nudo RJ, Jenkins WM, Merzenich MM (1990). Repetitive microstimulation alters the cortical representation of movements in adult rats. Somatosens Mot Res.

[pbio-0030362-b40] Recanzone GH, Merzenich MM, Dinse HR (1992). Expansion of the cortical representation of a specific skin field in primary somatosensory cortex by intracortical microstimulation. Cereb Cortex.

[pbio-0030362-b41] Dinse HR, Godde B, Hilger T, Haupt SS, Spengler F (1997). Short-term functional plasticity of cortical and thalamic sensory representations and its implication for information processing. Adv Neurol.

[pbio-0030362-b42] Dinse HR, Böhmer G, Schütz A, Miller R (2002). Plastic-adaptive properties of cortical areas. Cortical areas: Unity and diversity.

[pbio-0030362-b43] Godde B, Leonhardt R, Cords SM, Dinse HR (2002). Plasticity of orientation preference maps in the visual cortex of adult cats. Proc Natl Acad Sci U S A.

[pbio-0030362-b44] Stoney SD, Thompson WD, Asanuma H (1968). Excitation of pyramidal tract cells by intracortical microstimulation: Effective extent of stimulating current. J Neurophysiol.

[pbio-0030362-b45] Heusler P, Cebulla B, Boehmer G, Dinse HR (2000). A repetitive intracortical microstimulation pattern induces long-lasting synaptic depression in brain slices of the rat primary somatosensory cortex. Exp Brain Res.

[pbio-0030362-b46] Dinse HR, Kalisch T, Ragert P, Tegenthoff M (2005). Improving haptic perfromance in normal and impaired human populations through unattended activation-based learning. Transaction Appl Perc.

[pbio-0030362-b47] Karhu J, Tesche CD (1999). Simultaneous early processing of sensory input in human primary (SI) and secondary (SII) somatosensory cortices. J Neurophysiol.

[pbio-0030362-b48] Wolters A, Sandbrink F, Schlottmann A, Kunesch E, Stefan K (2003). A temporally asymmetric Hebbian rule governing plasticity in the human motor cortex. J Neurophysiol.

[pbio-0030362-b49] Boroojerdi B, Prager A, Muellbacher W, Cohen LG (2000). Reduction of human visual cortex excitability using 1-Hz transcranial magnetic stimulation. Neurology.

[pbio-0030362-b50] Muellbacher W, Ziemann U, Boroojerdi B, Hallett M (2000). Effects of low-frequency transcranial magnetic stimulation on motor excitability and basic motor behavior. Clin Neurophysiol.

[pbio-0030362-b51] Berardelli A, Inghilleri M, Rothwell JC, Romeo S, Curra A (1998). Facilitation of muscle evoked responses after repetitive cortical stimulation in man. Exp Brain Res.

[pbio-0030362-b52] Peinemann A, Lehner C, Mentschel C, Munchau A, Conrad B (2000). Subthreshold 5-Hz repetitive transcranial magnetic stimulation of the human primary motor cortex reduces intracortical paired-pulse inhibition. Neurosci Lett.

[pbio-0030362-b53] Wu T, Sommer M, Tergau F, Paulus W (2000). Lasting influence of repetitive transcranial magnetic stimulation on intracortical excitability in human subjects. Neurosci Lett.

[pbio-0030362-b54] Di Lazzaro V, Oliviero A, Mazzone P, Pilato F, Saturno E (2002). Short-term reduction of intracortical inhibition in the human motor cortex induced by repetitive transcranial magnetic stimulation. Exp Brain Res.

[pbio-0030362-b55] Cohen LG, Bandinelli S, Sato S, Kufta C, Hallett M (1991). Attenuation in detection of somatosensory stimuli by transcranial magnetic stimulation. Electroencephalogr Clin Neurophysiol.

[pbio-0030362-b56] Seyal M, Siddiqui I, Hundal NS (1997). Suppression of spatial localization of a cutaneous stimulus following transcranial magnetic pulse stimulation of the sensorimotor cortex. Electroencephalogr Clin Neurophysiol.

[pbio-0030362-b57] Knecht S, Ellger T, Breitenstein C, Bernd Ringelstein E, Henningsen H (2003). Changing cortical excitability with low-frequency transcranial magnetic stimulation can induce sustained disruption of tactile perception. Biol Psychiatry.

[pbio-0030362-b58] Satow T, Mima T, Yamamoto J, Oga T, Begum T (2003). Short-lasting impairment of tactile perception by 0.9Hz-rTMS of the sensorimotor cortex. Neurology.

[pbio-0030362-b59] Drager B, Breitenstein C, Helmke U, Kamping S, Knecht S (2004). Specific and nonspecific effects of transcranial magnetic stimulation on picture-word verification. Eur J Neurosci.

[pbio-0030362-b60] Herron CE, Lester RA, Coan EJ, Collingridge GL (1986). Frequency-dependent involvement of NMDA receptors in the hippocampus: A novel synaptic mechanism. Nature.

[pbio-0030362-b61] Stanton PK, Sejnowski TJ (1989). Associative long-term depression in the hippocampus induced by hebbian covariance. Nature.

[pbio-0030362-b62] Bliss TV, Collingridge GL (1993). A synaptic model of memory: Long-term potentiation in the hippocampus. Nature.

[pbio-0030362-b63] Morris RG, Moser EI, Riedel G, Martin SJ, Sandin J (2003). Elements of a neurobiological theory of the hippocampus: The role of activity-dependent synaptic plasticity in memory. Philos Trans R Soc Lond B Biol Sci.

[pbio-0030362-b64] Lynch MA (2004). Long-term potentiation and memory. Physiol Rev.

[pbio-0030362-b65] Huang YZ, Edwards MJ, Rounis E, Bhatia KP, Rothwell JC (2005). Theta burst stimulation of the human motor cortex. Neuron.

[pbio-0030362-b66] Karni A, Sagi D (1991). Where practice makes perfect in texture discrimination: Evidence for primary visual cortex plasticity. Proc Natl Acad Sci U S A.

[pbio-0030362-b67] Crist RE, Li W, Gilbert CD (2001). Learning to see: Experience and attention in primary visual cortex. Nat Neurosci.

[pbio-0030362-b68] Schoups A, Vogels R, Qian N, Orban G (2001). Practising orientation identification improves orientation coding in V1 neurons. Nature.

[pbio-0030362-b69] Fahle M, Poggio T (2002). Perceptual learning.

[pbio-0030362-b70] Harris JA, Miniussi C, Harris IM, Diamond ME (2002). Transient storage of a tactile memory trace in primary somatosensory cortex. J Neurosci.

[pbio-0030362-b71] Ragert P, Dinse HR, Pleger B, Wilimzig C, Frombach E (2003). Combination of 5 Hz repetitive transcranial magnetic stimulation (rTMS) and tactile coactivation boosts tactile discrimination in humans. Neurosci Lett.

[pbio-0030362-b72] Kammer T (1999). Phosphenes and transient scotomas induced by magnetic stimulation of the occipital lobe: Their topographic relationship. Neuropsychologia.

